# A simple algorithm to estimate genetic variance in an animal threshold model using Bayesian inference

**DOI:** 10.1186/1297-9686-42-29

**Published:** 2010-07-22

**Authors:** Jørgen Ødegård, Theo HE Meuwissen, Bjørg Heringstad, Per Madsen

**Affiliations:** 1 Nofima Marin, P.O. Box 5010, NO-1432 Ås, Norway; 2 Department of Animal and Aquacultural Sciences, Norwegian University of Life Sciences, P.O. Box 5003, NO-1432 Ås, Norway; 3 Geno Breeding and A. I. Association, P.O. Box 5003, NO-1432 Ås, Norway; 4 Department of Genetics and Biotechnology, Faculty of Agricultural Sciences, Aarhus University, DK-8830, Tjele, Denmark

## Abstract

**Background:**

In the genetic analysis of binary traits with one observation per animal, animal threshold models frequently give biased heritability estimates. In some cases, this problem can be circumvented by fitting sire- or sire-dam models. However, these models are not appropriate in cases where individual records exist on parents. Therefore, the aim of our study was to develop a new Gibbs sampling algorithm for a proper estimation of genetic (co)variance components within an animal threshold model framework.

**Methods:**

In the proposed algorithm, individuals are classified as either "informative" or "non-informative" with respect to genetic (co)variance components. The "non-informative" individuals are characterized by their Mendelian sampling deviations (deviance from the mid-parent mean) being completely confounded with a single residual on the underlying liability scale. For threshold models, residual variance on the underlying scale is not identifiable. Hence, variance of fully confounded Mendelian sampling deviations cannot be identified either, but can be inferred from the between-family variation. In the new algorithm, breeding values are sampled as in a standard animal model using the full relationship matrix, but genetic (co)variance components are inferred from the sampled breeding values and relationships between "informative" individuals (usually parents) only. The latter is analogous to a sire-dam model (in cases with no individual records on the parents).

**Results:**

When applied to simulated data sets, the standard animal threshold model failed to produce useful results since samples of genetic variance always drifted towards infinity, while the new algorithm produced proper parameter estimates essentially identical to the results from a sire-dam model (given the fact that no individual records exist for the parents). Furthermore, the new algorithm showed much faster Markov chain mixing properties for genetic parameters (similar to the sire-dam model).

**Conclusions:**

The new algorithm to estimate genetic parameters via Gibbs sampling solves the bias problems typically occurring in animal threshold model analysis of binary traits with one observation per animal. Furthermore, the method considerably speeds up mixing properties of the Gibbs sampler with respect to genetic parameters, which would be an advantage of any linear or non-linear animal model.

## Background

Animal models are the most widely used for the genetic evaluation of Gaussian traits. An animal model can account for non-random mating and complex data structures including phenotypes of both parents and offspring, which is likely to cause bias in sire- or sire-dam models. Furthermore, in practical selection, animal models are necessary for optimal selection among individuals with their own phenotypic information, and the animal model is thus the most relevant from an animal breeding perspective [1]. However, animal threshold models applied to cross-sectional binary data (one observation per individual) have been shown to give a biased estimation of genetic parameters, particularly in the presence of numerous fixed effect classes [2-4], and genetic variance has been shown to "blow up" to unreasonably high values when using Markov chain Monte Carlo methods (e.g., the Gibbs sampler). Treating contemporary groups and other relevant effects as "random" or increasing the number of observations per subclass may to some extent overcome these problems, but is not optimal and cannot be considered as an universal solution [3]. Instead, binary data are often modeled through sire or sire-dam threshold models, but, as stated above, this is not appropriate for all data structures, as parents with individual records may cause bias in estimating genetic parameters. Another widely used option is to use linear models, even though this is statistically inappropriate for binary data. Still, predicted breeding values from linear and threshold models have shown good agreement in a number of studies [e.g., [5-7]]. The bias typically associated with animal threshold models should not be confused with general extreme-category problems (when all observations within a fixed category belong to one of the binary classes), as the latter may cause bias for threshold models in general.

The aim of this study was to develop an algorithm to estimate genetic (co)variance components using Bayesian inference via Gibbs sampling that solves the estimation problems commonly seen in cross-sectional animal threshold models. The proposed method is also applicable in other types of statistical models, and is generally expected to improve Markov chain mixing properties of the genetic parameters.

## Methods

In a standard threshold (probit) model, the observed binary records (*Y*_*ij*_) are assumed fully determined by an underlying liability (*λ*_*it*_), such that:

i.e., the threshold value is set to zero. In matrix notation the threshold animal model can be written as:

where: **λ **= vector of all *λ*_*ij*_, **β **= vector of "fixed" effects, **a **= vector of random additive genetic effects of all individuals, **e **= vector of random residuals, and **X **and **Z **are the appropriate incidence matrices.  and , where **A **is the additive genetic relationship matrix of all individuals, **I**_**n **_is an identity matrix with dimension equal to number of records, and  and  are the additive genetic and residual variances, respectively. As usual for probit threshold models,  is restricted to be 1.

In the following, the vector **a **will be split in two sub-vectors: , where **a**_**p **_includes breeding values of all parents (informative), while **a**_**np **_includes breeding values of non-parents (non-informative). The breeding values of non-parent animals can also be written as: **a**_**np **_= ½**Z**_**p**_**a**_**p **_+ **m**, where **Z**_**p **_is an incidence matrix assigning parents to each individual and  (in the absence of inbreeding) is a vector of Mendelian sampling deviations. The prior density of breeding values can be expressed as:

where **A**_**sd **_is the additive relationship matrix for sires and dams. As Mendelian sampling deviations of non-parents are independent of the mid-parent means, they can only be inferred from the phenotype(s) on the animal itself. For cross-sectional binary data, both the corresponding residual and the Mendelian sampling deviation are inferred from a single liability only, and are thus not identifiable (on the likelihood level) and completely confounded. Hence, these two parameters can be combined as in a reduced animal model:

where . Furthermore as **e **and e* are not identifiable on the likelihood level, the corresponding variances (and thus also the variances of **m **and **a**_**np**_) cannot be identified either. In threshold models, it is common to restrict  to be 1, and similar restrictions may also be imposed on the variance of **m**, which can be restricted to  (half the current sample of the genetic variance).

In the new algorithm, breeding values of all individuals (conditional on covariance components and liabilities) are sampled as in a standard animal model. However, the method differs from the standard animal model with respect to sampling of genetic covariance components. In a standard model, genetic variance is sampled conditional on all breeding values (both **a**_**p **_and **a**_**np**_). Assuming an univariate model, the fully conditional density of the genetic variance is:

which is in the form of a scale inverted chi-square distribution with *q *(dimension of **A**) degrees of freedom and scale parameter (**a'A^-1^a**), where . However, as stated above, the breeding values included in **a**_**np **_are not informative with respect to additive genetic variance. In the new algorithm, sampling of genetic (co)variance components is therefore solely based on parental breeding values (**a**_**p**_), i.e., between-family variation, and the fully conditional density of genetic variance is thus:

which is in the form of a scale inverted chi-square distribution with *r *(number of parents) degrees of freedom and scale parameter , where **Ma **= **a**_**p **_is a vector of parent breeding values (which has identifiable variance), **M **is the appropriate (r × q) design matrix (identifying "informative" individuals), and **A**_**sd **_is the additive relationship matrix for the individuals included in **a**_**p **_(parents). Note also that the fully conditional density of the new algorithm is proportional to the fully conditional density of additive genetic (sire-dam) variance under a sire-dam model:

where  and **u **is a vector of additive genetic sire and dam effects (transmitting abilities).

Although shown in a univariate setting, the proposed algorithm can easily be extended to a multivariate model.

### Simulation study

A total of 10 replicate data sets were generated. Each data set consisted of 2000 individuals with one binary observation each. Animals with data were the offspring of 100 sires and 200 dams, i.e., each sire was mated with two dams and each dam was mated with one sire (typical design for aquaculture breeding schemes), and full-sib families consisted of 10 offspring. For simplicity, sires and dams were assumed unrelated. Underlying liabilities were sampled following standard assumptions (i.e., residual variance was set to 1 and the threshold value set to zero), assuming a heritability of 0.20 (i.e., additive genetic variance was  = 0.25). The expected incidence rate was 50% (i.e., overall mean on the liability scale was zero).

Ideally, the effect of the new algorithm should be investigated in datasets where estimation problems are likely to occur, e.g., in datasets having a high number of fixed effect classes. Since the simulated fixed structure was rather simple (including an overall mean only), more complex fixed structures were imposed in the subsequent analysis by randomly assigning observations to 80 different fixed effect dummy classes (25 observations per class). Hence, numerous fixed effects were estimated in the subsequent analysis, although no real difference existed between them. To avoid creating additional extreme-category problems, the generated fixed effect structure of each replicate was checked to ensure that both binary categories were represented within each fixed class.

The MATLAB^® ^http://www.mathworks.com software was used to generate and analyze data. All models included a Gibbs sampling chain of 25,000 rounds (5000 burn-in and 20,000 sampling rounds). Sire-dam models are widely used and considered appropriate to analyze such data (as no parents had individual records). Therefore, for comparison purposes the data sets were analyzed using two animal threshold models (standard and new algorithm) and a sire-dam threshold model.

#### Animal model (Anim)

with parameters as described above. Here, the vector **β **had 80 subclasses. Two different Gibbs sampling schemes were used:

*AnimA*: A standard Gibbs sampling scheme, using common algorithms for all parameters (including the genetic variance). For each round of the Gibbs sampler, heritability was calculated as: .

*AnimB*: Same model as AnimA, except that additive genetic variance was sampled using the new algorithm as described above. Heritability was calculated as in model AnimA.

#### Sire-dam model (SireDam)

where **u **is a vector of additive genetic effects of sires and dams (transmitting abilities), **Z**_**p **_is an appropriate incidence matrix for parents and the other parameters are as described above. Here, .

## Results

**Figure 1:**
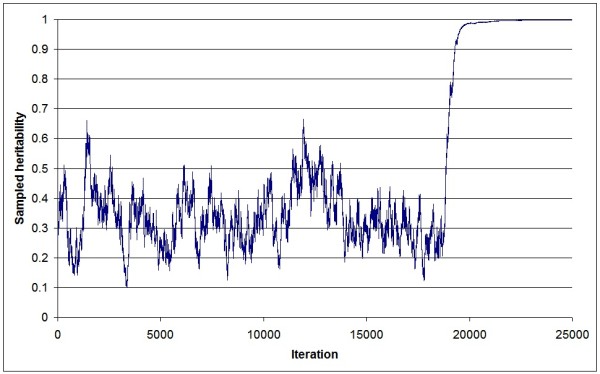
**Trace plot of sampled heritability values of the AnimA threshold model**. All samples from a Gibbs sampling chain (replicate 1) consisting of 25,000 iterations are shown; genetic variance is sampled based on the standard algorithm

**Figure 2:**
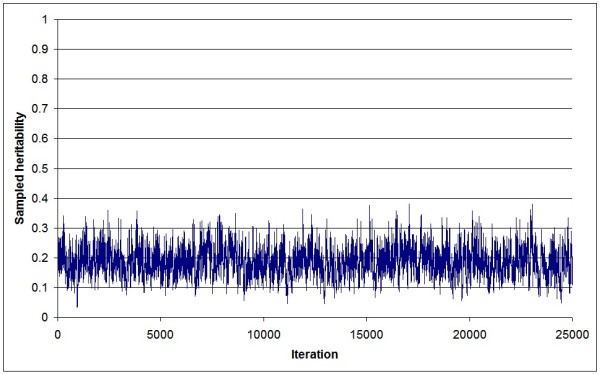
**Trace plot of sampled heritability values of the AnimB threshold model**. All samples from a Gibbs sampling chain (replicate 1) consisting of 25,000 iterations are shown; genetic variance is sampled based on the new algorithm

**Figure 3:**
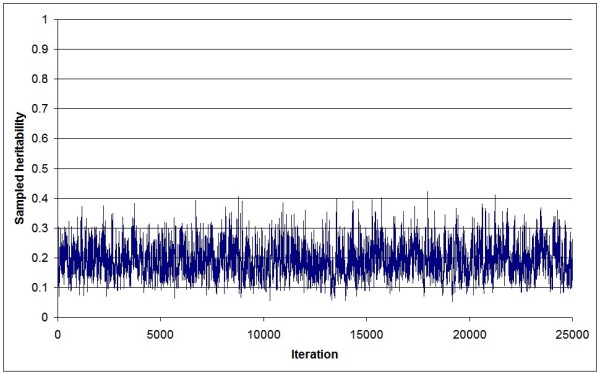
**Trace plot of sampled heritability values of the SireDam threshold model**. All samples from a Gibbs sampling chain (replicate 1) consisting of 25,000 iterations are shown; genetic variance is sampled based on the standard algorithm

Figure 1 shows a trace plot of heritability samples from a standard animal model (AnimA) applied to a simulated dataset (replicate 1). The plot clearly illustrates poor mixing, and a Gibbs sampler that never "converges". Heritability samples approach unity towards the end of the sampling period, i.e., genetic variance approaches infinity. Figure 2 shows the corresponding trace plot of heritability samples obtained for the same dataset using an identical animal model, but where the genetic variance was sampled using the new algorithm (AnimB). Here, mixing was much faster, and the samples were within a reasonable parameter space, given an input heritability of 0.20. Finally, the same dataset was analyzed using a standard sire-dam model (SireDam), and very similar results (Figure 3) as AnimB were obtained (after appropriate rescaling).

Averaged over the 10 replicates, posterior means of the heritability (Table 1) for AnimB and SireDam were both 0.25 (ranging from 0.17 to 0.37). Within each replicate, the two models gave almost identical posterior means of heritability (mean absolute difference was 3 * 10^-3^). Still, some replicates of both AnimB and SireDam showed a tendency towards overestimated heritability. However, as the same results were obtained with both the SireDam and the AnimB models, this bias was not related to the new algorithm, but more likely resulted from problems with the data structure (e.g., number of records and fixed effect structure). In contrast, the standard animal model (AnimA) resulted in severely overestimated heritabilities, as genetic variance drifted towards infinity for all replicates (as exemplified in Figure 1). The AnimA model was also analyzed with a Metropolis-Hastings random walk algorithm to estimate genetic variance, where breeding values were integrated out of the likelihood. However, the latter method gave essentially the same result as previously seen for AnimA with genetic variance drifting towards infinity (results not shown).

**Table 1:** Posterior means and standard deviations of underlying heritability for a binary trait^1^

Replicate	AnimB	SireDam
1	0.184 (0.048)	0.189 (0.052)
2	0.203 (0.049)	0.207 (0.055)
3	0.248 (0.047)	0.243 (0.056)
4	0.256 (0.051)	0.252 (0.056)
5	0.325 (0.052)	0.325 (0.060)
6	0.370 (0.051)	0.368 (0.063)
7	0.179 (0.047)	0.174 (0.052)
8	0.213 (0.048)	0.218 (0.053)
9	0.329 (0.053)	0.330 (0.061)
10	0.210 (0.050)	0.205 (0.056)

Average	0.251	0.251
Input parameter	0.200	0.200

^1^The two models presented are an animal threshold model using the new algorithm for sampling of additive genetic variance (AnimB) and a standard sire-dam threshold model (SireDam); posterior standard deviations are given in parentheses.

Although similar posterior means of heritability were obtained using the AnimB and SireDam models, posterior standard deviations of the heritability were generally slightly higher for the SireDam model (Table 1). However, a preliminary analysis showed that this discrepancy was largely removed if residual variance of the SireDam model was restricted to , rather than  = 1 (results not shown).

## Discussion

Severe bias was observed for a cross-sectional standard animal threshold model (AnimA) when applied to small data sets with unfavorable fixed effect structures (deliberately chosen to create estimation problems). For all 10 replicates, the AnimA model resulted in genetic variance drifting towards infinity (both using standard Gibbs sampling and a random walk algorithm). However, the problems associated with animal models were solved by employing the new algorithm to sample additive genetic variance (AnimB), resulting in essentially identical heritability estimates as an appropriate sire-dam threshold model (SireDam). Both AnimB and SireDam models showed a tendency towards overestimated heritabilities in some replicates, which may be explained by the small and unfavorably structured datasets. Consequently, apparent differences between the fixed effect classes may be incorrectly accounted for by the model, resulting in overestimated heritability. Nevertheless, this problem was equally expressed in the AnimB and SireDam models, and thus it is not a result of the new algorithm.

The bias typically seen in animal threshold models (AnimA) may be explained by an interaction between the random and fixed effects of the model, i.e., preliminary analyses revealed that all models were seemingly appropriate for a simple fixed effect structure (overall mean only). Hence, the problem has some similarities with classical extreme-category problems (ECP), which occur when all observations within a fixed class belong to the same binary category (which was not the case here). Typically, ECP are avoided by defining the relevant effects as random. In a cross-sectional threshold model, the animal classes are defined as random, but the classes are always extreme (one observation per animal). Hence, our hypothesis is that, given unknown genetic variance, classical animal models may still cause ECP in some cases. For increasing genetic variance, the random animal effects will increasingly resemble fixed effects, potentially resulting in ECP at some point during the Markov chain. The risk of this is likely to increase with the number of fixed effect classes in the data (as this would increase uncertainty of genetic parameters). As observed in this study, the sampled genetic variance in the AnimA model varies substantially until it eventually reaches such large values that the chain seemingly enters an absorbing state (Figure 1). Furthermore, the putative genetic variance has different impacts on parental and non-parental breeding values, which may explain the better results obtained with AnimB (and SireDam). Given high putative genetic variance, non-parental breeding values would be increasingly confounded with the associated (and extreme) liabilities, while parental breeding values would be based on the liabilities of multiple offspring (normally on both sides of the threshold), making the latter less extreme (and closer to the true values). Hence, based on AnimB and SireDam, sampled genetic variance is likely to quickly stabilize at appropriate values.

The results indicate that the AnimB model gives slightly lower posterior standard deviations for the heritability compared with the SireDam model. This may be explained by differences in the definition of phenotypic variance of liability in the two models. For an animal threshold model, the phenotypic variance is: , and the heritability is thus  while for a sire-dam threshold model, the phenotypic variance is: , and the heritability is . Hence, a proportional change in the genetic (sire-dam) variance of the two models will have a larger effect on the heritability in a sire-dam model. However, we do know that the residual variance of a sire-dam model (in the absence of inbreeding) necessarily includes half the additive genetic variance , and the residual variance may thus be restricted to: , with the corresponding heritability being: , which is analogous to the heritability of an animal model. As expected, preliminary analyses showed that the latter type of restriction largely removed the discrepancies between posterior standard deviations of heritability for the SireDam and AnimB models.

The proposed algorithm is not only relevant in threshold model analyses of cross-sectional binary data (one observation per individual), it is also of particular relevance in the analysis of time-until-event and sequential binary data. In the latter type of data, repeated records may exist for each individual, but one of the binary categories (e.g., dead) terminates the recording period. In the presence of time-dependent or stage-specific fixed effects, variances of individual random effects (e.g., permanent environment and Mendelian sampling terms) are non-identifiable for such traits [8], which may lead to bias in animal-, sire- or sire-dam models, either as a result of biased estimates of additive genetic variance components (animal model) and/or as a result of lacking ability to account for covariance among observations on the same individual (sire- and sire-dam models). Given that genetic (co)variance components can be accurately estimated, an animal model will properly account for genetic covariance between repeated observations on the same individual. However, in sequential binary data, an animal model (including AnimB) will be unable to identify covariance structures explained by individual permanent environmental effects.

Across traits, Mendelian sampling deviations of non-parents are, in most cases, completely confounded with either residuals (cross-sectional data) or permanent environmental effects (longitudinal data). Thus, non-parent individuals can usually be regarded as "non-informative" under sampling of additive genetic variance without any loss of information. In preliminary analyses, we also applied the AnimA and AnimB models to data sets with repeated (non-sequential) binary records for each individual, assuming the existence of permanent environmental effects. As expected, both models gave essentially identical results, but the AnimB model showed better Markov chain mixing properties (results not shown). Hence, even in cases where a standard animal model is expected to give unbiased results (e.g., Gaussian traits, or repeated, non-sequential binary data), applying the new algorithm is expected to improve mixing of additive genetic parameters (being similar to a sire-dam model).

In this study, all parents had multiple offspring with data and were therefore considered "informative" with respect to additive genetic (co)variance components. However, this would not be true for parents/ancestors having only a single descendant with data. Therefore, if present, such individuals should be defined as "non-informative" in sampling of additive genetic (co)variance components.

The new algorithm to estimate genetic (co)variance components is now implemented as an option in the Gibbs sampling module of the DMU statistical software package [9], where it is adapted to handle multivariate genetic analyses including binary, ordered categorical and Gaussian traits.

## Conclusions

The new Gibbs sampling algorithm (AnimB) allows appropriate estimation of genetic (co)variance components for animal threshold models. In contrast, a standard animal threshold model (AnimA) applied to the same data sets resulted in samples of genetic variance drifting towards infinity. Given that the data sets could be appropriately analyzed (no parental phenotypes) with a sire-dam threshold model (SireDam), the SireDam and AnimB models yielded essentially identical results. Furthermore, AnimB is also expected to improve Markov chain mixing properties of animal models in general, and may therefore be advantageous in all types of animal models using Gibbs sampling. The new algorithm is now implemented as an option in the Gibbs sampling module of the DMU software package for multivariate genetic analysis.

## Competing interests

The authors declare that they have no competing interests.

## Authors' contributions

JØ derived the theory, generated simulated data sets, performed the statistical analyses and wrote the manuscript. PM implemented the methodology in the DMU statistical software package. All authors took part in discussions, made input to the writing and read and approved the final manuscript.

## References

[B1] Moreno C Sorensen D García-Cortés LA Varona L Altarriba J On biased inferences about variance components in the binary threshold model Genet Sel Evol 1997 29 145 160 10.1186/1297-9686-29-2-145

[B2] Hoeschele I Tier B Estimation of variance components of threshold characters by marginal posterior modes and means via Gibbs sampling Genet Sel Evol 1995 27 519 540 10.1186/1297-9686-27-6-519

[B3] Luo MF Boettcher PJ Schaeffer LR Dekkers JCM Bayesian inference for categorical traits with an application to variance component estimation J Dairy Sci 2001 84 694 704 10.3168/jds.S0022-0302(01)74524-9 11286423

[B4] Stock K Distl O Hoeschele I Influence of priors in Bayesian estimation of genetic parameters for multivariate threshold models using Gibbs sampling Genet Sel Evol 2007 39 123 137 10.1186/1297-9686-39-2-123 17306197 PMC2682833

[B5] Ødegård J Olesen I Gjerde B Klemetsdal G Evaluation of statistical models for genetic analysis of challenge-test data on ISA resistance in Atlantic salmon (Salmo salar): Prediction of progeny survival Aquaculture 2007 266 70 76 10.1016/j.aquaculture.2007.02.012

[B6] Ødegård J Kettunen Præbel A Sommer AI Heritability of resistance to viral nervous necrosis in Atlantic cod (*Gadus morhua *L.) Aquaculture 2010 300 59 64 10.1016/j.aquaculture.2010.01.006

[B7] Heringstad B Rekaya R Gianola D Klemetsdal G Weigel KA Genetic change for clinical mastitis in Norwegian cattle: a threshold model analysis J Dairy Sci 2003 86 369 375 10.3168/jds.S0022-0302(03)73615-7 12613880

[B8] Visscher PM Thompson R Yazdi H Hill WG Brotherstone S Genetic analysis of longevity data in the UK: present practice and considerations for the future INTERBULL Bulletin 1999 16 22

[B9] Madsen P Jensen J DMU: a user's guide. A package for analysing multivariate mixed models 2007 University of Aarhus, Faculty of Agricultural Sciences, Department of Animal Breeding and Genetics Version 6, release 4.7

